# Role of the Genetic Background in Resistance to Plant Viruses

**DOI:** 10.3390/ijms19102856

**Published:** 2018-09-20

**Authors:** Jean-Luc Gallois, Benoît Moury, Sylvie German-Retana

**Affiliations:** 1 INRA-UR 1052, Génétique et Amélioration des Fruits et Légumes (GAFL), Domaine St Maurice, CS 60094, F-84143 Montfavet CEDEX, France; jean-luc.gallois@inra.fr; 2 INRA, Unité Pathologie Végétale, 67 Allée des Chênes, Domaine Saint Maurice, CS 60094, F-84143 Montfavet CEDEX, France; benoit.moury@inra.fr; 3 UMR 1332, Biologie du Fruit et Pathologie, INRA, Univ. Bordeaux, 71 Av. E. Bourlaux, CS 20032, 33882 Villenave d’Ornon CEDEX, France

**Keywords:** plant, virus, resistance, durability, genetic background, Quantitative Trait Loci, epistasis

## Abstract

In view of major economic problems caused by viruses, the development of genetically resistant crops is critical for breeders but remains limited by the evolution of resistance-breaking virus mutants. During the plant breeding process, the introgression of traits from Crop Wild Relatives results in a dramatic change of the genetic background that can alter the resistance efficiency or durability. Here, we conducted a meta-analysis on 19 Quantitative Trait Locus (QTL) studies of resistance to viruses in plants. Frequent epistatic effects between resistance genes indicate that a large part of the resistance phenotype, conferred by a given QTL, depends on the genetic background. We next reviewed the different resistance mechanisms in plants to survey at which stage the genetic background could impact resistance or durability. We propose that the genetic background may impair effector-triggered dominant resistances at several stages by tinkering the NB-LRR (Nucleotide Binding-Leucine-Rich Repeats) response pathway. In contrast, effects on recessive resistances by loss-of-susceptibility—such as eIF4E-based resistances—are more likely to rely on gene redundancy among the multigene family of host susceptibility factors. Finally, we show how the genetic background is likely to shape the evolution of resistance-breaking isolates and propose how to take this into account in order to breed plants with increased resistance durability to viruses.

## 1. Introduction

In a given organism, the expression and effect of genes rely on a multitude of interactions within the genome and with the environment. Notably, phenotypes associated with specific genes are known to have effects of varying intensity depending on the variation of additional genetic factors, a phenomenon well characterized in the development of most organisms [1,2]. Several studies showed how similar mutations affecting genes are associated with very different, if not contradictory, phenotypes, and how these discrepancies rely ultimately on the so-called “genetic background”. Interestingly, the analysis of the genetic background effect can be a key to unravel so far uncharacterized mechanisms or redundancy effects among genes with an emphasis on developmental processes [3]. Genetic background has been defined as “the entire genetic and genomic context of an organism; the complete genotype of an organism across all loci” [1]. In the view of a plant breeder, who aims at producing elite crops by introducing multiple traits originating from Crop Wild Relatives (CWR) into crops [4,5], it is understandable that the whole breeding process will be linked with numerous introgressions into the crop genome, therefore resulting in profound changes in the genetic background. For example, it is estimated that nearly one quarter of the tomato genome is made of introgressed genome from CWR into cultivated tomato, including a 50 Mb introgression on chromosome nine, associated with a *Tm2*^a^ resistance, to the tomato mosaic virus, originating from *Solanum peruvianum* [6]. More recently, it was shown that this latter introgression has been associated with profound changes by linkage with genes affecting metabolism [7]. It seems highly likely that such “hitch-hiking” effects may impact as well responses to biotic/abiotic stresses among other plant features.

Genetic resistances to pest and pathogens represent a large proportion of traits sought after by breeding programs. Plants have evolved sophisticated resistance mechanisms to pathogens, including viruses that can be responsible for heavy crop losses. Very briefly, those mechanisms can be categorized as resistance associated with pathogen recognition, followed by defense induction and resistance by the loss-of-susceptibility affecting plant factors on which the pathogen relies for infection. In addition, quantitative resistance relying on Quantitative Trait Loci (QTL) can decrease pathogen traits related to infection efficiency or can modulate the efficiency of major-effect resistance genes [8,9]. As a result of the breeding process, resistances that have been isolated as simple genetic traits or/and QTL in CWR are transferred into crops.

The durability of a resistance can be defined as, “the persistence of resistance efficiency when resistant cultivars are used over long periods, on large surfaces and in the presence of the target pathogen” [10]. Low resistance durability is the consequence of appearance and the increase in the frequency of Resistance-Breaking (RB) variants within virus populations. The genetic changes required for a pathogen to overcome plant resistance mechanisms and the effects of such changes on its fitness are key determinants of resistance durability [11].

In this review, we discuss how the plant genetic background can affect the efficiency and durability of genetic resistance to viruses and what the potential underlying molecular mechanisms are. By taking the point of view of the breeders, we will not here discuss the role of the pathogen genetic background on resistance breaking (for example, [12,13]). The role of the genetic background will be discussed in the light of what is known about the resistance mechanisms. We will pay attention to what has also been characterized on the resistance durability to other pathogens, as this may hint at future research directions.

## 2. Genetic Background Matters in Resistance to Viruses: Lessons from QTL Analyses

The strategy based on the transfer of a resistance gene from a donor genotype or CWR into a recipient elite cultivar, dates back to Holmes’ work on tobacco and pepper tobamovirus resistance in the 1930s [14,15]. Since then, backcross programs aiming to perform such transfers have been very successfully undertaken by plant breeders. In the biotechnology era, transgenesis has even shown that resistance gene transfers to distant plant species, for which crosses are not feasible, could also be efficient. This indicates that all, or a very large part, of the resistance phenotype is expressed after these transfers and suggests a little effect of the genetic background of the recipient cultivar on resistance expression.

Notwithstanding, there have been few reports of major-effect resistance genes with incomplete penetrance [16,17,18,19,20,21]. Penetrance can be defined as the proportion of individuals that carry a particular gene and express the expected phenotype—here being resistance [1]. Penetrance is said “complete” when 100% of individuals carrying the gene express the phenotype, and “incomplete”, with different possible degrees. The penetrance of a particular resistance gene can be potentially affected by genetic background effects, but also by environmental effects. In the case of plant resistance to pathogens, such environmental effects may include changes of pathogenicity, i.e., aggressiveness or resistance-breaking properties in the pathogen population [17]. Unfortunately, these different kinds of effects have rarely been disentangled. However, when climatic conditions and pathogen populations are homogeneous and stable, incomplete penetrance may be attributable to genetic background effects. Gene expressivity, i.e., the extent of phenotypic expression in an individual carrying a given gene controlling this phenotype [1], could also provide a measure of the effect of the genetic background and/or of the environment on a resistance gene. However, measures of expressivity of major-effect resistance genes have rarely been conducted. It should be noted that the term penetrance, and even more, expressivity, are used much more in the field of animal biology than in plant biology.

A more frequent and precise source of data to quantify the role of the genetic background on resistance is the genetic analysis of quantitative resistance (QTL analyses). Basically, QTL analyses rely on additive genetic models, where the effects of different resistance loci cumulate in an additive manner to determine the overall resistance level. More complex genetic models also include non-additive pairwise effects between loci, i.e., epistatic effects. Epistasis occurs when the effect of a particular allele on the resistance level is different when associated to different alleles present at one or more other loci in the genetic background. Epistatic effects can thus be measured by the extent of the departure from a purely additive model. Consequently, the abundance and effect of epistasis involving resistance loci provides a measure of the effect of the genetic background on resistance genes.

We have collected published QTL analyses on virus resistance with a focus on those that incorporated epistasis analyses (Table 1). These 19 studies identified a large number of QTL with additive effects and these additive QTL usually explain a large part of inherited resistance variability (i.e., broad-sense heritability). In such situations, the effect of a resistance gene or locus (QTL) will simply add to that of the genetic background.

It should be noted that the majority of studies analyzed epistatic effects only among pairs of QTLs that were previously shown to display additive effects, with the exception of nine studies of virus resistance in pepper (Table 1A). From these nine studies, we can observe that: (i) Some QTL have epistatic effects but no detectable additive effects, (ii) the number of additive QTL is higher than the number of epistatic QTL (30 vs. 18) but this difference is not statistically significant (*p* = 0.22; *χ*^2^ test), and (iii) the effects of additive QTL and of epistases between pairs of QTL on the resistance level are similar (22% and 20% on average, respectively). If we consider the 19 QTL studies (Table 1A,B), 67 additive QTL were detected in total. Among these, 30 (45%) also displayed significant epistatic relationships with one or more of the other additive QTL. Epistatic effects were smaller than additive effects (15% vs. 21%). Epistatic effects are observed for QTL that display small additive effects (proportion of phenotypic variance explained by the QTL *R*^2^ < 0.25), but also for major-effect QTL (*R*^2^ > 0.25) or even QTL that have strong effects and could be almost considered as major-effect genes (*R*^2^ > 0.60). Moreover, if we categorize the additive QTL into “purely additive” and “both additive and epistatic”, those in the second category have a significantly higher additive effect on the resistance phenotype than those in the first category (mean *R*^2^ of 17.4% and 26% for “purely additive” and “both additive and epistatic” QTL, respectively; *p*-value = 0.005; Monte-Carlo simulations). This suggests that a major-effect QTL could be more sensitive to genetic background effects than minor-effect QTL. This partly supports Chandler et al. who argued that, in general, alleles of moderate phenotypic effects, that are intermediate between major-effect alleles and minor-effect QTLs, show the greatest sensitivity to genetic background [1].

The overall picture that emerges from these studies is that epistatic effects (and by extension genetic background effects) (i) are not rare, (ii) involve fewer loci than additive effects, and (iii) have smaller effects on the resistance phenotype than additive effects. However, the abundance of epistasis among resistance QTL and the intensity of epistatic effects are probably underestimated. First, epistasis has mainly been studied between QTL previously shown to display additive effects and QTL showing only epistatic effects have rarely been looked for (Table 1). Second, all studies of epistasis have focused on pairwise effects between loci and interactions between more than two loci have not been explored.

Though widespread, QTL analyses usually do not provide insights into the mechanisms involved in the genetic background effects on resistance genes. This is mainly because most of these QTLs have not been cloned [9]. Mechanistic studies have been performed mostly on major-effect resistance genes. In a few instances, resistance QTL can correspond to particular alleles of major-effect genes. For example, the resistance QTL *pvr2* and *pvr6* were shown to encode the translation initiation factors eIF4E and eIFiso4E, respectively [22,38,39]. Overall, similar background effects can be expected on the two categories of resistance listed above, effector-triggered resistance, and resistance by loss-of-susceptibility.

## 3. Effector-Triggered Dominant Resistances: Efficiency and Durability Depend on Genetic Background

Active resistance processes to viruses have been characterized in many plant species as relying on the specific recognition of pathogen proteins, called Avirulence factors (Avr) or effectors. These mechanisms are known as Effector Triggered Immunity or ETI (see reference [40]). The resistance process is mediated by *R*-genes, encoded by the plant genomes, that code for proteins containing Nucleotide Binding Domains and Leucine-Rich Repeats (NB-LRR). NB-LRR factors are encoded by large families of genes and are associated with resistance to all kinds of pathogens and pests affecting plants (oomycetes, fungi, bacteria, insects, and nematodes, etc.). In the frame of the current review, we will only focus on NB-LRR associated with resistance to viruses, a topic that has been recently reviewed elsewhere [41]. The NB-LRR protein may interact directly or indirectly with the viral effector, which triggers a complex signaling pathway ultimately resulting in the activation of the resistance response. ETI often ultimately results in a Hypersensitive Response (HR), an induced localized plant cell death at the site of penetration of the pathogen, highly recognizable by necrosis, and resulting in the pathogen’s death. In some cases, Extreme Resistance (ER) has been described at the cell level with no apparent necrosis. In all cases, ETI includes very complex signaling cascades involving protein phosphorylations, involvement of phytohormones, and activation of specific transcription factors, resulting in the expression of Pathogenesis Related (PR) proteins.

*R* genes controlling dominant resistance, isolated from CWR, are favored by breeders and have been introgressed into many elite crop cultivars [8,42]. Many *R* genes have been cloned and the corresponding *Avr* factors identified [41], but significantly, *R* genes have been routinely selected in breeding schemes based on resistance phenotype or marker-assisted selection without the formal identification of the underlying gene. While NB-LRR are seen as the major determinant of ETI, introgressing them to new genetic background can undermine their function. While a number of *R* genes retain their effectiveness when transgenically introduced into heterologous plant species belonging to the same family, as exemplified by the transfer of *Rx* in *Nicotiana* species [43], NB-LRR genes tend not to function properly when transferred to plants of a different family [44]. This may be due to lack of appropriate protein partners. Conversely, because NB-LRR proteins have to be tightly regulated, introgression into a new genetic background can destabilize this repression, leading to spontaneous tissue necrosis (reviewed in [45]).

*R*-based resistance-breaking is usually associated with mutations in the viral Avirulence factors, resulting in their non-recognition by the *R* gene [46,47,48,49]. The resistance durability is closely linked to (i) the fitness cost associated with the RB mutations and (ii) the opportunity of the mutation to arise [50,51,52,53,54]. Pleiotropic effects of resistance-breaking mutations may result in across-host fitness trade-offs, known as resistance-breaking (or virulence) costs. For different pathosystems involving *R*-mediated resistance and viruses, resistance-breaking costs were analyzed through the monitoring of within-host components of the viral fitness [55,56,57,58]. Those studies showed that the resistance-breaking mutants were usually less competitive than the parental isolates in susceptible plants. Duff-Farrier et al. [59] showed that the durability of the *Rx*-mediated resistance differs according to the genetic backgrounds. The potato *Rx* gene provides resistance against the *Pepino mosaic virus* (PepMV) in tomato (*Solanum lycopersicum*). The authors showed that two point mutations in the avirulence factor (coat protein; CP) of PepMV conferred *Rx* breaking in *S. lycopersicum*, whereas they did not confer *Rx* breaking in *N. tabacum* and only one of them allowed for *Rx* breaking in *N. benthamiana*, suggesting that the durability of Rx-mediated resistance may depend on the genetic background in which it is present. Outside those extreme effects of the genetic background on resistance, more subtle effects have been described concerning *R*-based resistance in conjunction with introgression. Deng et al described how the resistance to the bacterium *Xanthomonas oryzae* pv. *oryzae* (Xoo), associated with membrane-localized LRR protein encoded by *Xa3/Xa26*, is more efficient in the *Oryza sativa* ssp. japonica rice background than in the *O. sativa* ssp. indica one [60], a fact that is associated with a higher expression of *Xa3*/*Xa26*. One QTL associated with differences in resistance [61] is associated with a defense-responsive WRKY transcription factor. They noticed how the japonica background is associated with a higher expression of the *WRKY45* allele 1 that might contribute to an increased response to *Xa3/Xa26*-mediated response [60]. Similarly, genetic background has been shown to be instrumental in the resistance mediated by *Me1* and *Me3 R* genes in pepper toward nematodes: introgression of those genes into a susceptible background made it more likely for the plant to be attacked by the parasite than when they were introgressed into a partially resistant background [62].

**Figure 1:**
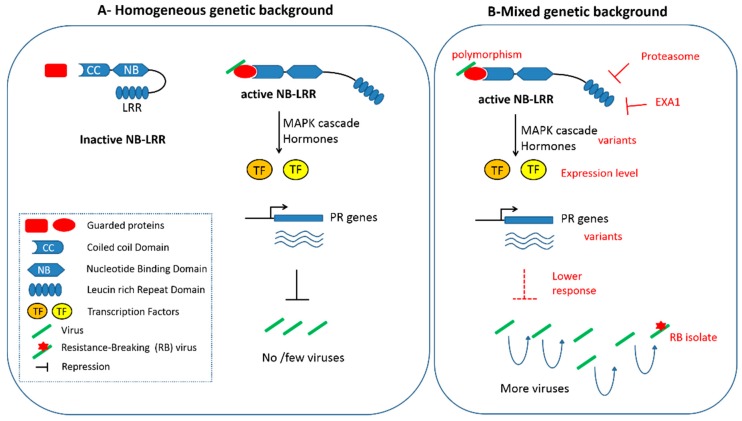
Potential effects of the genetic background on a NB-LRR based resistance to a viral pathogen. (**A**) classical representation of the guard model. On the left side, NB-LRR remains inactive in plants unchallenged by virus (represented by green rods). On the right side, virus-activation of NB-LRR resulting in a signaling cascade involving MAP Kinases and hormones, Transcription Factors (TF) activation of Pathogenesis-Related (PR) genes and ultimately, repression (or suppression) of the virus in the plant. (**B**) Potential regulations of the response cascade susceptible to act at each stage of the signaling response are indicated in red (see accompanying text for details). Those variations may decrease the efficiency of the response, therefore resulting in a higher accumulation of viruses in the plant, allowing the development of resistance breaking (RB) isolates, through mutations represented by a red star.

Recent studies have highlighted molecular factors affecting NB-LRR accumulation and consequently resistance expression (Figure 1). Among them, the glycine-tyrosine-phenylalanine (GYF) domain protein EXA1 has been identified as a negative regulator of NB-LRR protein accumulation, and as a result, limits plant immunity [63]. Other factors, such as molecular chaperones and the ubiquitin-mediated proteasome system have been shown to affect the NB-LRR folding, accumulation, or turnover [64,65]. However, network analysis involving *Arabidopsis* mutants impaired in different branches of the resistance pathway (namely Jasmonate, Ethylene, Phytoalexin Deficient 4 and Salicylate) shows how those pathways are connected and buffered [66]. This allows us to maintain a robust response to pathogens, even if one branch of the signaling pathway, downstream of the *R* gene, is impaired [67]. Altogether, those studies show that variability among those factors, as well as among downstream regulatory pathways between genetic backgrounds, could modulate the resistance response. This in turn could directly affect how pathogens can subsist in plants, therefore allowing selection of resistance-breaking variants and jeopardizing the resistance durability.

Besides genetic background, strictly speaking, the role played by epigenetic mechanisms in plant-pathogen interactions has gained interest during the last years [68]. Indeed, DNA methylation and demethylation, various histone modifications, noncoding small-interfering RNA (sRNA)-mediated transcriptional gene silencing, and posttranscriptional gene silencing (PTGS) were shown to be implicated in the control of plant defense against pathogens [68,69]. While only a few studies associate chromatin modification to plant innate immunity [70], histone modification was shown to be directly involved in chromatin remodeling and transcriptional control of a subset of *R* genes in Arabidopsis [71]. However, most of those studies refer to fungal and bacterial pathogens. Concerning resistance to plant viruses, the majority of studies describe epigenetic mechanisms involving RNA molecules (sRNA) that were shown to naturally protect plants from viruses, which can be both inducers and targets of PTGS. Those epigenetic regulations involved in plant-virus interactions, including virus-induced transgenerationally inherited epigenetic modifications, were recently discussed by Baulcombe and Dean [72]. Sharma et al. highlighted, in particular, that siRNA-mediated methylation of viral DNA confers resistance to various plant DNA viruses, including geminiviruses (family Geminiviridae) [73]. Epigenetic modifications were shown to be involved in virus resistance via siRNA-mediated RNA-directed DNA methylation by Yadav and Chattopadhyay [74]. The authors reported that a soybean variety resistant to a geminivirus, showed a rapid degradation of viral RNAs, in comparison to a susceptible variety, due to the fact that viral DNA is targeted at the transcriptional level, while virus-derived transcripts are targeted by posttranscriptional silencing. This is a nice example of viral genome methylation as an epigenetic defense against geminiviruses.

At the plant level, DNA methylation could be involved with resistance to viruses. Indeed, Zhao et al. studied the DNA methylation polymorphism in flue-cured tobacco accessions and candidate markers for *Tobacco mosaic virus* (TMV) resistance [75]. In particular, they conducted experiments to analyze the DNA methylation pattern within TMV-resistant and TMV-susceptible tobacco groups using the methylation-sensitive amplified polymorphism (MSAP) technique. Their results showed that three polymorphic sites (MSAP fragments) were significantly correlated with TMV resistance [75]. Recently, a possible role of the methylation of the resistance gene *CTV.20* in response to *Citrus tristeza virus* (genus Closterovirus, family *Closteroviridae*) infection was suggested [76]. A hypermethylation of the *CTV.20* gene was observed in resistant *Poncirus trifoliata* and tolerant to carrizo citrange (derived from a cross *C. sinensis* L. Osb. × *P. trifoliata* L. Raf.), either healthy or infected, while a partial demethylation was detected in susceptible sweet (*Citrus sinensis* L. Osb.) and sour orange (*Citrus aurantium* L.) following CTV infection [76]. Altogether, those studies confirmed that epigenetic mechanisms can play a role in virus resistance.

## 4. Resistances Associated with Mutations in Susceptibility Factors: Redundancy, an Ambivalent Factor That Impacts Resistance Durability

The completion of the viral cycle results from a complex interplay between virus and host-encoded factors, also called susceptibility factors. In this scheme, absence or non-adequacy of a single susceptibility factor leads to full or partial recessive resistance to viruses [77]. The first demonstration of this concept of loss-of-susceptibility resistance genes in crops came out through the identification of eukaryotic initiation factors as key players in plant-potyvirus interactions [38,78,79,80]. Since then, antiviral recessive resistance genes in model plants and several crop species were identified and have been reviewed [81]. Recently, the genetic manipulation of eukaryotic initiation factors using CRISPR/Cas9 technology allowed to confer virus resistance or lower susceptibility in important crops such as cucumber, rice, and cassava [82,83,84]. Moreover, a proof-of-concept study in *Arabidopsis* showed that eIF4E genes could be engineered to copy natural resistance alleles and generate resistance at no yield loss [85].

Often, those susceptibility factors have been shown to be encoded by small multigenic families. Functional redundancy among those multiple paralogs of a susceptibility factor can play either in favor or against resistance or its durability. In the former case, the redundancy enables diversification of the susceptibility factors and the plant fitness is not affected whereas viruses lose the copy they can use. In the latter case, viruses can use multiple copies and therefore can break down the resistance. The positive role of host gene redundancy on resistance has been exemplified in Chinese cabbage (*Brassica rapa* cv pekinensis) [86]. Redundancy of copies of eukaryotic translation initiation factors in Chinese cabbage was shown to arise from the mis-splicing of *eIFiso4E* gene, enabling diversification and resulting in the plant being able to evade virus infection. Indeed, when the copy that *Turnip mosaic virus* (TuMV) normally uses in Chinese cabbage is non-functional for both plant and virus, there is no apparent disadvantage for the plant. The durability of the resistance is dependent upon the virus not being able to mutate in order to hijack other eIFiso4E orthologs. In the lettuce (*Lactuca sativa*)/*Lettuce mosaic virus* (LMV) pathosystem, the *mo1* resistance conferred by mutations in the eIF4E copy has largely remained durable in the field, despite the ability to easily select RB LMV variants in laboratory experiments [13]. The durability of the *mo1* lettuce resistance could be explained by a combination of factors, including the nature and fitness costs associated with these mutations, the fact that LMV is not able to use the eIFiso4E isoform in lettuce [78], but also the geographical structure of LMV populations [13].

**Figure 2:**
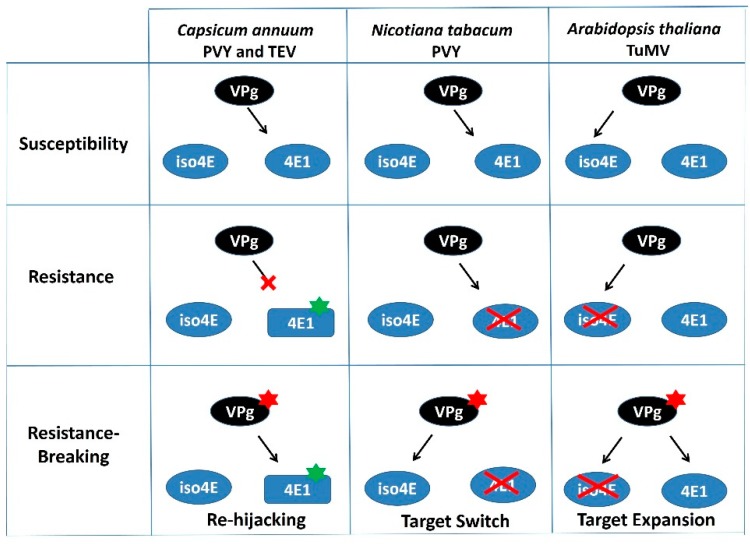
Different Resistance-Breaking pathways for potyviruses in plants. Potyviruses are represented by their VPg (main virulent determinant for eIF4E-mediated resistance). Two plant translation initiation factors are represented: eIF4E1 (4E1) and its isoform eIFiso4E (iso4E). Mutations affecting eIF4E1 or the viral VPg are represented by green and red stars, respectively. KO mutants for *eIF4E* or *eIFiso4E* are crossed out. The figure is a schematic representation of resistance-breaking strategies in *Capsicum annuum*, *Nicotiana tabacum* and *Arabidopsis thaliana* as depicted in References [85,87,88], respectively. Briefly, in *Capsicum annuum*, mutation in the viral VPg allows the virus to hijack the resistant eIF4E1 protein; in *Nicotiana benthamiana*, it allows the virus to recruit eIFiso4E while losing its initial ability to recruit eIF4E1; finally, in *Arabidopsis thaliana*, it allows the virus to recruit both eIF4E1 and eIFiso4E.

In opposition, viruses can evolve towards resistance breaking through the acquisition of mutations that either restore compatible interactions with the mutated host susceptibility factor, as seen in pepper (*Capsicum annuum*) with the *pvr2*/*eIF4E1* resistance breakdown [87], or that possibly allow PVY to switch from eIF4E to the isoform eIFiso4E, as recently suggested in tobacco (*Nicotiana tabacum*) [88] (Figure 2). Lastly, Bastet et al. [85] showed how mutation within the TuMV-VPg, associated with loss-of-function (Knock-Out) of *eIFiso4E* (hereafter named *eifiso4e*) resistance-breaking [89], expanded the virus ability to recruit both isoforms, eIFiso4E and eIF4E1. As a result, resistance to the RB isolate could only be attained when the broken-down *eifiso4e* resistance allele was combined with an *eif4e1* resistance allele. It is highly likely that by integrating an *eif4e1* resistance allele in combination with *eifiso4e*, a higher durability of the latter gene should be achieved, although this remains to be tested. All these examples, taken from various plants and pathosystems, illustrate how redundancy among eIF4E-coding genes may reduce eIF4E-based resistance durability by making other eIF4E factors available to viruses [90]. It also suggests how the elusive genetic background effect can, in some cases, be narrowed down to simple redundancy effects involving one or a small number of genes.

**Figure 3:**
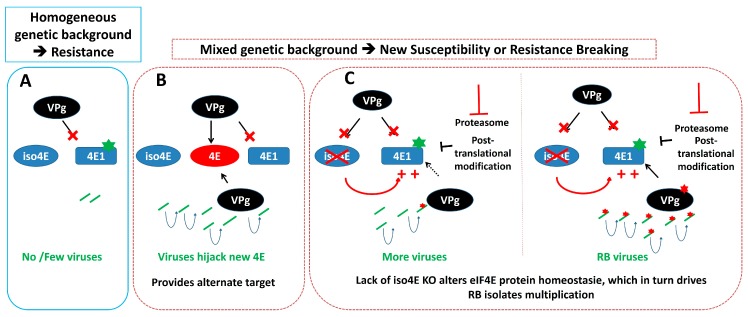
Potential effects of the genetic background on a eIF4E-based resistance to a viral pathogen. (**A**) Simple representation of eIF4E-based resistance. (**B**) Potential modulation of this resistance (or resistance breaking) in mixed genetic background through the presence of an additional eIF4E factor (indicated in red), or (**C**), by regulation of eIF4E accumulation level through feedback mechanisms or modulation of the eIF4E level regulation. Increased eIF4E accumulation allows minimal virus replication and results in the emergence of RB isolates. Mutations affecting the host eIF4E1 or the viral VPg are represented by green and red stars, respectively. The ‘plus’ sign indicates an increased accumulation of eIF4E1. The ‘cross mark’ indicates that the virus cannot recruit the corresponding eIF4E copy.

In relation to the impact of protein accumulation on NB-LRR-based resistance mechanisms (see above), similar considerations could be put forward on *eIF4E*-based resistance (Figure 3). Regulatory feedbacks among different translation initiation factors isoforms have been shown earlier [91]. Moreover, eIFiso4E protein accumulation has been shown to be regulated by the ubiquitin proteasome system [92] and members of the eIF4E family are known to be post-translationally regulated [90]. Interestingly, in tomato (*Solanum lycopersicum*), a comparison between a natural functional eIF4E resistance allele and an engineered loss-of-function KO allele, unveiled an unexpected regulatory process between the members of the eIF4E family, impairing the effectiveness of a resistance strategy based on eIF4E-KO [93]. Therefore, any genetic factor regulating the accumulation of plant susceptibility proteins could play a role on resistance durability. In this regard, it is very interesting that in pepper, the major durability QTL identified, associated with the *eIF4E1 pvr2^3^* allele overcoming is located in the region of a natural loss-of-function allele of *eIFiso4E*, suggesting that similar regulatory mechanisms could be at stake (see below). It is also interesting to note that mutations affecting the glycine-tyrosine-phenylalanine (GYF) domain protein EXA1, identified as a negative regulator of NB-LRR protein accumulation (see above), is also involved in resistance to *Plantago asiatica mosaic virus* (PlAMV), a potexvirus [94]. The authors suggested that this resistance might be associated with the direct regulation of the susceptibility factors eIF4E and eIF4G. It remains to be determined whether EXA1, which also contains a conserved eIF4E-binding motif, could function by upregulating unidentified NB-LRR or through indirect effect on eIF4E.

## 5. The Genetic Background Drives the Evolution of Resistance-Breaking Isolates

### 5.1. Role Played by Minor Genes (Quantitative Trait Loci or QTL) in the Level and the Durability of the Resistance Conferred by a Major Gene

The presence of QTL, besides the major-effect resistance gene, was shown to impact the durability of resistance in different pathosystems (plant–virus/fungus/nematode) [95,96,97]. The emergence of RB variants overcoming the major resistance gene was very rare or did not occur in the plant genotypes combining the major resistance gene in a quantitatively resistant genetic background, but increased in a susceptible genetic background. In pepper, Quenouille et al. identified plant QTL for a trait related to the frequency of the emergence of RB variants of PVY overcoming major resistance alleles at the *eIF4E1 pvr2* locus [26,98]. In particular, they showed that all QTL involved in viral accumulation were also involved in the RB frequency of the major resistance gene *pvr2*. Interestingly and unexpectedly, the QTL allele with the largest effect on the decrease of RB frequency came from the susceptible parent of the progeny. In contrast, the minor-effect QTL alleles came from the resistant parent. This means that transfer of a resistance gene in the genetic background of an elite cultivar could either increase or decrease resistance durability. Later on, the same authors evaluated a larger collection of pepper landraces, representing the worldwide genetic diversity, for its ability to modulate the breakdown frequency by PVY of *pvr2* [99]. The RB-mutations in the virus differed between plant genotypes, indicating differential selection effects exerted on the virus population by the different genetic backgrounds. The RB frequency was positively correlated with the level of virus accumulation, confirming the impact of quantitative resistance loci on resistance durability [99].

In tobacco, combining a major PVY resistance gene with another gene involved in viral accumulation has proved to enhance *eIF4E va-*mediated resistance durability [100]. The authors suggested that combining the *va* gene, responsible for limiting potyvirus cell-to-cell movement, with another *va2* locus, involved in limiting virus accumulation, could increase the durability of *va*-mediated resistance. The correlation between increased virus accumulation and reduced durability of the resistance is consistent with a simple explanation where the virus has a higher probability of accumulating mutations associated with RB [26,98,99]. Following their generation, the fate of resistance-breaking variants depends not only on their accumulation (fitness) but also on the genetic drift that randomly changes the frequencies of viral variants from generation to generation. The success of the emergence of the RB variants (and as a consequence, the decrease of resistance durability) depends, in particular, on the viral effective population size (*Ne*), defined “as the number of individuals passing their genes to the next generation” [27]. During plant infection, the viral population undergoes bottlenecks, leading to drastic reductions in *Ne,* and potentially, to the loss of the fittest variants. Therefore, plant genetic factors may affect *Ne*, and slowing down pathogen evolution could increase resistance durability. Tamisier et al. showed that *Ne* at the viral inoculation step is a diversified and highly heritable trait among a pepper population [27], and one of the QTL controlling *Ne* may also decrease virus adaptation to pvr2-mediated resistance [27]. Accordingly, Rousseau et al. showed that the frequency of *pvr2* resistance breakdown was determined by complex interactions between virus *Ne*, the level of resistance efficiency, and the intensity of selection exerted by the plant on the virus population [101].

### 5.2. Role Played by the Genetic Background in the Modulation of the Genetic Drift and Selection within Virus Populations

Owing to their large population size and short generation time, viruses have a great potential to quickly evolve and adapt under selection pressures [102]. Host factors (including genetic background), in combination with environmental factors, likely affect virus evolution and adaptation, helping viruses to invade new hosts and potentially form more virulent strains [50,103,104]. The durability of deployed resistance genes ultimately depends on the overall evolutionary potential of viruses (which is determined by the genetic structure of viral populations), on the functional flexibility of the pathogen avirulence factors targeted by the resistance gene(s), and on ecological effects which may increase or slow down the emergence of resistance-breaking isolates [103]. Diversity in viral species has been described previously as a mechanism to avoid host resistance or as a reservoir to maintain variants with selective advantages in other environments, and has been correlated with the ability to infect numerous hosts [105]. In particular, it is generally assumed that weeds and wild hosts have a broader genetic diversity than crop species, and therefore allow for the establishment of viral populations with a higher degree of genetic variability, potentially including resistance-breaking variants that can be selected at a higher frequency [106]. There are many examples of damaging viruses emerging into cultivated crops from native ecosystems, and it has been argued that “the viral genetic variability contained in reservoir populations is the most important genetic determinant of viral emergence” [103]. Weeds, where viral populations are under selective constraints that are potentially different from those in crops, could contribute to the emergence of RB variants for crops. Due to the maintenance of virus inoculum sources for long periods in the field and the possibility of hosting mixed infections, weeds could play an important role in generating virus variability by mutation, recombination, and/or reassortment, representing a stepping-stone in moving towards resistance breaking. Garcia-Andres et al. [107], provided evidence of the relevance of the wild species *S. nigrum* as a reservoir of begomoviruses that causes epidemics of *Tomato yellow leaf curl* virus (TYLCD) and of recombination as a force driving their evolution. Jaag and Nagy [108] showed that environmental and host factors could play an important role in viral RNA recombination, one of the major driving forces in RNA virus evolution [103]. A recent example of the emergence of a resistance-breaking virus that may have resulted from host-switching, is the description of a new resistance-breaking tobamovirus, an Israeli isolate of *Tomato brown rugose fruit virus (*ToBRFV), that is able to overcome *Tm-2*-mediated resistance in tomato [109].

The complexity of host range expansion and therefore the difficulty of predicting the resistance-breaking in crops, was highlighted by Moreno-Perez et al. [110]. The authors showed that the effects on the virus fitness of different mutations in *Pepper mild mottle virus* (PMMoV) coat protein, that result in overcoming *L* gene resistance in pepper, depend on the host genotype and on the type (single or mixed) of infection. The authors showed that the RB mutations may be neutral, costly, or favorable for the virus fitness depending on the genotype of susceptible hosts [110].

In a recent study, Rousseau et al. [111] hypothesized that breeding of plant varieties exposing viruses to stronger genetic drift, could slow virus adaptation and may represent an approach for increasing resistance durability. In this study, the authors combined high-throughput sequencing with experimental evolution in order to follow, within the host dynamics of five RB-variants of PVY in fifteen closely related pepper genotypes that carry the resistance gene *pvr2^3^*, but differ in their genetic backgrounds. Their results showed that “the viruses experienced considerable diversity in genetic drift regimes, depending on the host genotype”. Importantly, they showed that the genetic drift experienced by virus populations was a heritable plant trait.

### 5.3. Combining Recessive and Dominant Resistance Genes Sharing the Same Viral Effector Impacts Resistance-Breaking

Evolutionary constraints upon viral resistance-breaking determinant can be imposed by a trade-off between the breakdown of a recessive resistance gene and host viability regulated by a dominant resistance gene [50,104]. In the pea/*Clover yellow vein virus* (ClYVV) pathosystem, Atsumi et al. [104] showed that the recessive gene *cyv1* confers resistance to ClYVV and the major viral resistance-breaking determinant is the P3N-PIPO protein. After overcoming the *cyv1* resistance, the RB variants accumulate efficiently and produce a more abundant P3N-PIPO that is also a viral effector recognized by the dominant gene *Cyn1*, which induces systemic cell-death and the loss of host viability that is unfavorable for the virus. The authors propose that a trade-off for the virus in overcoming paired defense mechanisms may sustain the durability of resistance against ClYVV [50,104].

## 6. Concluding Remarks

In this review, we gathered scattered studies that have carried out a close examination of genetic background effects on plant resistance genes or QTLs. Most of these studies have focused on resistance efficiency, and only a few of them on resistance durability. However, this question is of high and generic interest because the effect of genetic background determines the transferability of resistance genes or QTL, from one species (or genotype) to another, and the predictability and efficiency of genetic progress during breeding for resistance. Indeed, in the case of strong background effects, a large part of resistance efficiency, durability, or the spectrum of action may be lost after the transfer. The examination of QTL analyses of plant resistance to viruses summarized in Table 1 and supports the view that the majority of detected QTL have an additive, and therefore, predictable effects on the resistance phenotype. However, QTL involved in epistatic relationships (i.e., background dependent) are also frequent and the mean effect of epistases on the variation of the resistance phenotype is quite high. However, the types of epistasis were rarely mentioned in these studies. Indeed, three kinds of epistases could be distinguished, with different outcomes for resistance. Positive epistasis is the most favorable case as it corresponds to a more-than-additive effect between resistance alleles at two QTL. Reciprocal sign epistasis is the least favorable case as the combination of resistance alleles at two QTL contributes to a higher susceptibility than the combination of two susceptibility alleles. Finally, negative epistasis is intermediate, showing a less-than-additive effect between resistance alleles at two QTL. It would therefore be interesting to characterize more precisely the types of epistasis between resistance QTL to gain insight into the mechanisms involved and the resistance benefit that we could expect from these epistases. As a consequence, complex and frequent interactions are expected between resistance genes or QTL and the genetic background.

A few mechanistic studies showed that the variability among molecular factors present in the genetic backgrounds can affect NB-LRR accumulation, turnover, and downstream regulatory pathways, and could consequently modulate the resistance response conferred by *R*-genes. This, in turn, could directly affect how pathogens can subsist in plants, therefore allowing for the selection of Resistance-Breaking variants and jeopardizing resistance durability. The redundancy for recessive resistances, associated with mutations in susceptibility factors, has also been shown to be an ambivalent factor impacting resistance durability. Indeed, functional redundancy among multiple paralogs of a susceptibility factor can play either in favor or against the resistance level or its durability. In the former case, the redundancy enables diversification of the susceptibility factors and the plant fitness is not affected, whereas, viruses lose the copy they can use. In the latter case, viruses can use multiple copies and therefore can break down the resistance. Furthermore, recent experimental viral evolution studies performed in genotypes carrying the same major-resistance gene (inducing a strong selection pression), but differing in the genetic drift they impose to the viral population, suggest that breeding of plant varieties exposing viruses to stronger genetic drift could slow virus adaptation and may represent an approach for increasing resistance durability. From an agronomic point of view, selecting varieties where the control of virus evolution can be done by manipulating the evolutionary forces acting on virus populations (including multiplying bottlenecks) could allow to achieve better resistance durability. More generally, since background effects are largely unpredictable and can be either positive or negative on the phenotypic expression of resistance genes or QTL, one can advise breeders: (1) To diversify the recipient genotypes where resistance genes are introgressed, but also, (2) to select directly for favorable backgrounds, like in recurrent selection programs that allow genome mixing between several genotypes.

## Figures and Tables

**Table 1:** Published data on virus resistance QTL in plants. (Only studies where epistases between resistance QTL have been looked for are indicated.)

Reference	Plant	Virus	Number of Additive QTL	Effect of Additive QTL (*R*^2^) ^a*,*b^	Number of Epistatic QTL	Effect of Epistatic QTL (*R*^2^) ^a^	Global QTL Effect (*R*^2^) ^a^	Broad-Sense Heritability
**A—Studies Where Epistases Have been Looked for in the Whole Genome**								
[22,23]	*Capsicum annuum* (pepper)	*Pepper veinal mottle virus* (PVMV)	4	10%, 62% (67%, 68%) ^c^	2 (non additive)	20%	76% (including epistasis)	95%
[22,23]	*Capsicum annuum* (pepper)	*Potato virus Y* (PVY)	6	10%, 10%, 10%, 12%, 25% (***33%***) ^c^	2 (1 non additive)	25%	66–71% (including epistasis)	90–96%
[24]	*Capsicum annuum* (pepper)	*Cucumber mosaic virus* (CMV)	2	19%, ***24%***	2 (1 non additive)	33%	57% (including epistases)	94%
[25]	*Capsicum annuum* (pepper)	CMV (strain N)	3	4%, 8%, 64%	2 (non additive)	29%	NA ^d^	NA
[25]	*Capsicum annuum* (pepper)	CMV (strain MES)	2	***13%***, 45%	2 (1 non additive)	25%	NA	NA
[26]	*Capsicum annuum* (pepper)	PVY (virus accumulation or AUDPC)	4	15%, 16%, 16%, 34%	0	-	34–44%	64–98%
[26]	*Capsicum annuum* (pepper)	PVY (frequency of resistance breakdown)	3	***9%***, ***13%***, ***40%***	4 (1 non additive)	11, 17, 19%	69% (including epistases)	87%
[27]	*Capsicum annuum* (pepper)	PVY	3	6%, ***26%***, ***35%***	2 (both also additive)	11%	58% (including epistasis)	93%
[27]	*Capsicum annuum* (pepper)	CMV	3	11%, ***22%***, ***31%***	2 (both also additive)	9%	51% (including epistasis)	98%
**B—Studies Where Epistases Have been Looked for Only Between QTL with Additive Effects**								
[28]	*Beta spp. x Beta vulgaris* (sugar beet)	*Beet yellows virus* (BYV)	3	4%, 7%, 11%	0	-	NA	NA
[29]	*Cucumis melo* (melon)	CMV-M6	4	5%, 10%, ***12%***, ***35%***	2	9%	78% (including epistasis)	NA
[30]	*Zea mays* (maize)	*Maize chlorotic dwarf virus* (MCDV)	4	3%, ***4%***, ***24%***, 25%	2	NA	55% (including epistasis)	87%
[31]	*Prunus armeniaca* (apricot)	*Plum pox virus* (PPV)	6	9%, ***14%***, 15%, ***16%***, 17%, ***56%***	3	NA	12–78% (including epistasis)	59–72%
[32]	*Phaseolus vulgaris* (common bean)	*Beet curly top virus* (BCTV)	2	***12%***, ***47%***	2	NA	24–53% ^e^	NA
[33]	*Arabidopsis thaliana*	PPV	3	7%, 23%, 66%	0	-	NA	92%
[34]	*Cucumis melo* (melon)	*Tomato leaf curl New Dehli virus* (ToLCNDV)	3	***13%***, ***18%***, ***67%***	3	3, 11%	NA	NA
[35]	*Zea mays* (maize)	*Sugarcane mosaic virus* (SCMV)	5	8%, 10%, ***20%***, ***28%***, ***56%***	3	7, 18, 29%	77% (including epistases)	77–94%
[36]	*Avena sativa* (oat)	*Barley yellow dwarf virus* (BYDV)	4	***6%***, 8%, ***9%***, ***36%***	3	3, 5%	50–58% (including epistases)	58%
[37]	*Triticum aestivum* (wheat)	*Wheat yellow mosaic virus* (WYMV)	3	5%, ***10%***, ***54%***	2	5%	34–57% (without epistasis)	84%

^a^ Part of the phenotypic variance explained by the QTL(s) (coefficient of determination *R*^2^). When different values were available for a given QTL, depending on the analysis method or dataset, the maximal value was indicated. ^b^ in bold and italics: QTL showing significant epistatic effects. ^c^ figures between parentheses correspond to QTL that have been identified with a model that considers the major QTL as a co-factor. Therefore, the effects (*R*^2^ values) of these QTL cannot be compared with those of the other QTL. ^d^ NA: Data not available. ^e^ It was not clear from that study if the epistasis was included or not.

## Abbreviations

QTL	Quantitative Trait Loci
RB	Resistance breaking
eIF4E	Eukaryotic translation initiation factor 4E
NB-LRR	Nucleotide-Binding site Leucine Rich Repeats
BCTV	*Beet curly top virus*
BYDV	*Barley yellow dwarf virus*
BYV	*Beet yellows virus*
ClYVV	*Clover yellow vein virus*
CMV	*Cucumber mosaic virus*
CTV	*Citrus tristeza virus*
LMV	*Lettuce mosaic virus*
MCDV	*Maize chlorotic dwarf virus*
PepMV	*Pepino mosaic virus*
PlAMV	*Plantago asiatica mosaic virus*
PMMoV	*Pepper mild mottle virus*
PPV	*Plum pox virus*
PVMV	*Pepper veinal mottle virus*
SCMV	*Sugarcane mosaic virus*
TEV	*Tobacco etch virus*
TMV	*Tobacco mosaic virus*
ToBRFV	*Tomato brown rugose fruit virus*
ToLCNDV	*Tomato leaf curl New Dehli virus*
TuMV	*Turnip mosaic virus*
TYLCV	*Tomato yellow leaf curl virus*
WYMV	*Wheat yellow mosaic virus*
